# 

**Published:** 1899-08

**Authors:** 


### CONTENTS:

SELECTIONS.

Article I.—Pulp Mummification. Theodore Soderberg........... 145

" II.—Open Maxillary Sinus with Concomitant Maxillary Sinusitis. Faneuil D. Weisse, M. D................ 150

" III.—What are the Best Materials for Filling Children's Teeth. C. Edmund Kells, Jr., D. D. S............. 162

" IV.—Systemic Remedies in Diseases of the Teeth. Leo Greenbaum, M. D., D. D. S......................... 169

" V.—Nervous Patients. Dr. Walter F. Lewis .............. 175

" VI.—Professional Demeanor. R. B. Gentle ................ 180

COMMUNICATED.

Why Should New Jersey Compel Dentists to Register Annually.... 183

Colorado State Dental Association.............................. 185

EDITORIAL, ETC.

A Decision Which May Cost Dentists Millions..................... 185

Will Fight the Decision......................................... 190

MONTHLY SUMMARY.

Experiments with Nirvanin...................................... 191

Treatment of Carbolic Acid Poisoning........................... 192

